# Cellular injury to 1- to 3+-year-old stems of *Camellia sinensis* by *Tuckerella japonica*

**DOI:** 10.1007/s10493-017-0181-3

**Published:** 2017-11-29

**Authors:** Diann S. Achor, Carl C. Childers, Michael E. Rogers

**Affiliations:** 1 University of Florida, Lake Alfred, FL USA; 2 Citrus Research and Education Center, University of Florida, Lake Alfred, FL USA; 3 Citrus Research and Education Center, University of Florida, 700 Experiment Station Road, Lake Alfred, FL 33850 USA

**Keywords:** Tuckerellidae, Plant feeding injury by mites, Peacock mites, Tetranychoidea

## Abstract

*Tuckerella japonica* Ehara (Acari: Tuckerellidae) feeds on predigested plant cells beneath exposed periderm tissue of 1- to 3+-year-old stems of *Camellia sinensis* (L.) O. Kuntze (Theaceae) where longitudinal bark splitting occurs. Control samples from these tissues were compared with areas fed upon by *T. japonica* adults and immatures to characterize types of cellular injury. Stylet diameters ranged from 1.6 to 2.3 µm and were consistent with observed stylet punctures in the stems. Mite saliva was injected along tracts within the cortical tissue and resulted in cell wall disruption, collapsed cells and, in older tissue, hyperplasia. The range of potential stylet penetration into plant tissues was from 92 to 150 µm. *Tuckerella japonica* injects saliva in the cortical tissues. The paired stylet lengths would allow for possible injection of saliva into the upper areas of phloem tissue but not in the cambium area of wood exposed by splitting of the outer epidermis.

## Introduction

The Tetranychidae, Tenuipalpidae and Eriophyoidea comprise a large group of mite species that feed mainly on higher plants and numerous species are of economic importance (Banerjee and Cranham 1985; Childers and Derrick 2003; de Lillo and Skoracka 2010; Leigh 1985; McMurtry 1985). Some species of Tenuipalpidae and Eriophyidae are vectors of a number of viral diseases many of which cause significant economic losses to cultivated plants (Oldfield and Proeseler 1996; Childers and Derrick 2003; Rodrigues et al. 2016; Malagnini et al. 2016). Several eriophyoid mites are capable of producing galls or other abnormal growth on plants from their feeding (Westphal and Manson 1996; de Lillo and Monfreda 2004). A third group of eriophyoid mites can directly cause serious injury and damage to various plants from their feeding with the citrus rust mite, *Phyllocoptruta oleivora* (Ashmead), the pink citrus rust mite, *Aculops pelekassi* (Keifer), the grape rust mite, *Calepitimerus vitis* (Nalepa) and the apple rust mite, *Aculus schlectendali* (Nalepa) as examples (Childers 1994; Oldfield 1996; Khederi et al. 2014).

The Tetranychoidea and Eriophyoidea have piercing, paired stylets for their mouthparts but their morphology as well as feeding mechanisms and injuries differ (de Lillo et al. 2002). The citrus rust mite has shorter stylets (about 30 µm) with feeding injury confined to the epidermis of citrus fruit or leaves (Albrigo and McCoy 1974; McCoy and Albrigo 1975; Achor et al. 1990). Citrus rust mite feeding injury on citrus leaves and fruit resulted in evacuated epidermal cells or cell injury that led to necrosis of cellular contents. In contrast, stylet lengths of Tetranychidae have been reported to be about 100 µm in (Jeppson et al. 1975) and in an adult female of *Panonychus ulmi* (Koch), were recorded to be 118 µm (Avery and Briggs 1968). Their longer stylets permit penetrating into the mesophyll of the leaf and cortical layers of the fruit. Injury by *P. citri* (McGregor) to citrus leaves or fruit consisted of evacuated cells, sometimes leaving starch grains, or collapsed cells that were totally evacuated (Albrigo et al. 1981). In one study, feeding resulted in the collapse of the phloem in vascular bundles near stylet paths of the six-spotted spider mite, *Eotetranychus sexmaculatus* (Riley). The collapse was speculated to be caused by the extent of stylet penetration and mite saliva to the nearby photosynthate producing cells supporting the phloem rather than direct feeding in the phloem tissue (Albrigo et al. 1981).

Recovery has been observed in both types of feeding injury to leaves and fruit. In the case of the deeper feeding injury to leaves or fruit by the citrus red mite, *P*. *citri*, mesophyll cells surrounding the injured cells divided (hyperplasia) and replaced the cells that had been evacuated or collapsed due to feeding (Albrigo et al. 1981). In rust mite feeding, wound periderm has been observed to form due to injury to the epidermis (Achor et al. 1990).

*Brevipalpus californicus* (Banks), *B. phoenicis* (Geijskes) and *B. yothersi* Baker have been shown to be vectors of one or more viruses in plants (Chagas et al. 2003; Kondo et al. 2003; Kitajima et al. 2003; Rodrigues et al. 2003, 2016). In many instances, virus lesions from these non-systemic diseases occurred on stems as well as leaves and fruit, thus indicating that these mites fed on stems too. The diversity within this species complex is only beginning to be recognized (Sanchez-Velazquez et al. 2015).

*Tuckerella japonica* has been observed feeding on exposed green periderm tissues in the crevasses created by splitting bark on 1- to 3+-year-old woody stems of tea [*Camellia sinensis* (L.) O. Kuntze] (Childers et al. 2016). With the descriptions of typical injury and recovery patterns caused by the above groups of mites in mind, this study was designed to examine the crevasses in which *T. japonica* was found to define the type of injuries to underlying tissues. The following steps were taken to describe these injuries: (1) examine the crevasse tissues with scanning electron microscopy (SEM) to document punctures caused by penetration of the paired stylets, (2) use light and SEM to determine the length and diameter of the stylets and compare these measurements with punctures, (3) examine and describe the control tissues from crevasse areas of stems of *C. sinensis* extending from the inner vascular cambium layer to the outer developing cork periderm layer, (4) characterize cellular injury by *T. japonica* to those tissues, (5) examine the stylet track areas with a definitive stain, safranin, to determine if feeding tracks were from lignification or saliva, and (6) determine by comparison of stylet lengths to depth of discernible injury, whether stylet penetration by *T. japonica* in *C. sinensis* stems extended into the vascular cambium as suggested by Charles (2009).

Studies that show the locations and types of cellular injury to a host plant by a *Tuckerella* species are lacking. This paper reports on the injury incurred within 2-year-old stems of *C*. *sinensis* by stylet penetration and injection of saliva by *T. japonica*. The sampled varieties were not available for release as they are considered proprietary by the owner.

## Materials and methods

### Scanning electron microscopy (SEM) of mite feeding punctures

All 1- to 3-year-old stems for this study were taken between 2014 and 2016 from the Charleston Tea Plantation on Wadmalaw Island in Charleston, SC, USA. Approximately 30 to 100 cm lengths of 2-year-old stems of *C. sinensis* between 0.5 and 1.0 m and with longitudinal splitting of the bark were cut in the field with pruning shears, returned to the laboratory in Charleston, SC and examined using a stereomicroscope. Twenty 2 to 3 cm lengths of these stems showing evidence of *T. japonica* resting or feeding were cut using pruning shears. A second series of twenty 2 to 3 cm lengths of the same stems with no evidence of mite presence were taken each time for comparison.

Each piece was subsequently cut down the middle of the stem with a razor blade so that the rounded area where the mite(s) were located was evident. Each piece was immediately transferred into one of two vials (mites either present or absent) containing 3% glutaraldehyde in 0.1 M Sorenson’s buffer, pH 7.2 and kept on ice in transit from Charleston until further processing 24 h later in the laboratory at the Citrus Research and Education Center (CREC) in Lake Alfred, FL, USA. There the samples were washed 3 times in Sorenson’s buffer, post fixed in 2% osmium tetroxide in the same buffer and kept overnight at 4 °C. The following morning the samples were rinsed again in buffer, dehydrated in ethanol (10% steps, 10 min for each step) and dried using a Ladd Critical Point Dryer (Ladd Research Industries, Burlington, VT, USA). The dried samples were mounted on stubs, coated with gold/palladium using a Ladd Sputter Coater (Ladd Research Industries) studied and photographed with a Hitachi S530 Scanning Electron Microscope (Hitachi High-Technologies, Japan).

### Light microscopy (LM) and transmission electron microscopy (TEM) of plant tissues and mite injury from stylet penetration and injection of saliva

Two to 3 mm squared pieces of the above samples were prepared for TEM by placing them in 3% glutaraldehyde fixative and further processing them in the laboratory. The samples were rinsed in buffer, post-fixed in osmium as above, and rinsed again in buffer before dehydration in acetone (10% steps, 10 min for each step). Subsequently the samples were infiltrated in Spurr’s resin (Spurr 1969) over a 3 day period, placed in molds and hardened in an oven at 70 °C. One micrometer thick sections were prepared with a glass knife on a LKB Huxley Ultramicrotome (LKB Instruments, Sweden), stained with methylene blue/azure A and counter stained with basic fuchsin (Humphrey and Pittman 1974) for light microscopy. Sections were observed under an Olympus BX61 compound microscope (Cambridge Scientific Products, Watertown, MA, USA) and photographed using an OMAX CMOS 14mp digital camera. Ultrathin sections for TEM were prepared with a diamond knife on the same ultramicrotome, stained with 2% aqueous uranyl acetate, post-stained with Reynolds lead citrate (Reynolds 1963), and photographed using a Morgagni 268 transmission electron microscope (FEI Company, The Netherlands).

### Paraffin embedded samples for light microscopy (LM)

Samples of 1- and 2-year-old stems with visible splitting of the bark, containing tuckerellid mites or free of mites were collected in the Charleston Tea Plantation and placed in moist bags and kept on ice. The samples were then transported to CREC and the following day they were processed for light microscopy. Areas of obvious bark splitting were excised from the stems using razor blades and placed in FAA fixative (Formalin-Alcohol-Acetic Acid) overnight at 4 °C. The following day the samples were rinsed in 50% ethanol and dehydrated up to 100% ethanol (20% steps, 1 h for each step). The tissue was then cleared by infiltration with *tert*-Butanol (TBA) in 3 steps: 100% ethanol; TBA 3:1 1 h, 1:1 1 h, 1:3 1 h to overnight at room temperature. The tissue was subsequently infiltrated with liquid paraffin at 60 °C as follows: 3:1 TBA:liquid paraffin 6 h to overnight, 1:1 TBA:liquid paraffin overnight, followed by 3 changes in liquid paraffin each overnight at 60 °C. The samples were placed in molds, covered with liquid paraffin and placed in a refrigerator at 4 °C to solidify. Serial 10 µm sections of stem samples were prepared using a Leica RM 2155 rotary microtome (Leica Microsystems, Germany). The sections were mounted in water on glass slides and cured at 30 °C on a warming tray. The slides were stained with safranin/fast green (Sass 1958). With this staining, lignin, chromatin, cutin and saliva stain red, chloroplasts stain pink to red; and cellulose walls and cytoplasm stain green.

Control tissues were punctured with a 000 insect pin (0.25 mm diam) to rule out results due to mere mechanical injury by feeding. They were prepared from stem pieces of 1- or 2-year growth as above. Three separate samplings were made: five samples punctured and processed for microscopy at the time of collection, 5 punctured then processed 48 h later, and 5 more processed 3 days after the stem pieces were punctured. These were all processed for paraffin embedding using the above method. Light micrographs were prepared using the Olympus BX61 compound microscope as described above.

### Measurements of* Tuckerella japonica* stylets with LM and SEM

For LM measurements, the mites were collected and stored in 80% ethanol and slide-mounted later in Hoyer’s medium (Walter and Krantz 2009). Micrographs were taken using the Olympus BX61 compound microscope. The stylets were measured using Figi measurement software (Schindelin et al. 2012).

For SEM measurements, mites that were observed feeding were sprayed with chloroform to attempt to quickly kill them while the stylets were protracted and then transferred into 80% ethanol to preserve them. In the laboratory, the mites were dehydrated further through 3 changes of 100% ethanol then critical point dried as above. Each mite was then carefully mounted on a stub, sputter coated and observed as above. The stylet images were measured using Figi measurement software (Schindelin et al. 2012).

## Results and discussion

**Fig. 1:**
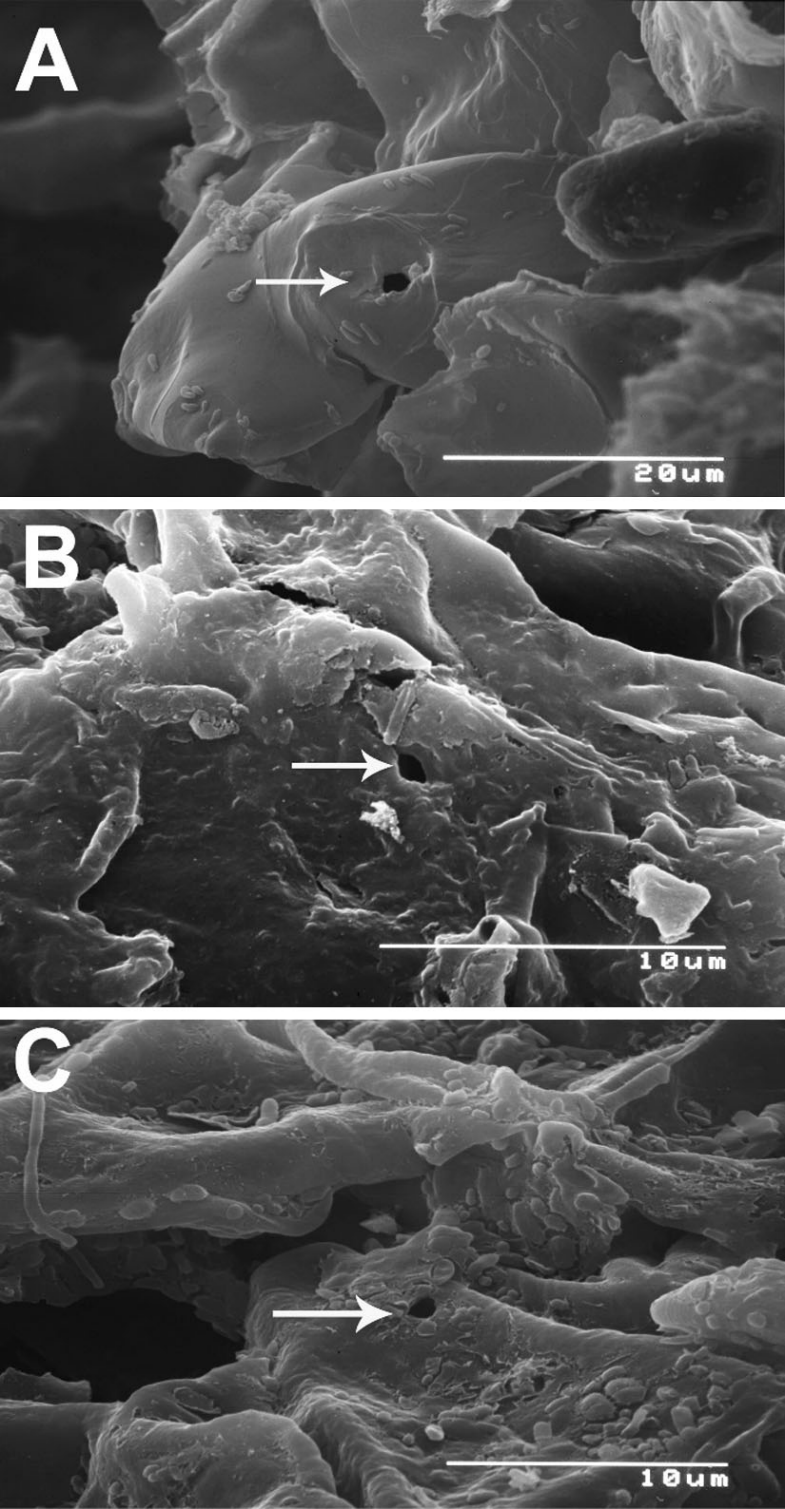
Scanning electron micrographs of *Tuckerella japonica* stylet punctures (white arrows) on exposed periderm cells in fissures revealed by the splitting of the outer cork periderm layers

Nine puncture measurements in the plant tissues ranged from 0.85 to 2.3 µm in diameter (Table 1). Stylet punctures by motile stages of *T. japonica* are shown in Fig. 1. The locations of these punctures were in green periderm tissues exposed by longitudinally split bark on 1- to 3+-year-old woody stems. As 1-year-old stems increased in size, there was increased longitudinal splitting of the periderm with 2-year-old stems having the most bark splitting (Bond 1942; Childers et al. 2016). Stylet lengths and diameters are presented in Table 1. Only 4 mites had stylet lengths greater than 70 µm when chloroform was applied. Measurements of these stylets from SEM photos were: 76.1, 81.6, 100.2, and 110 µm and partial retraction was believed to have occurred in each instance. The width of *T. japonica* stylets was consistent with the width of the SEM photos shown in Fig. 1. The entire stylets could be viewed through the transparent body of the mite with LM and these were the measurements for comparison.

**Table 1:** Measurements of one (single) or two (double) stylet diameter (µm) of immature and mature *Tuckerella japonica* and puncture width (µm) using scanning electron microscopy (SEM) and stylet section lengths (µm) of slide-mounted mites using light microscopy (LM) and SEM images

Stylet width (SEM)	Immatures	Matures
Single tip lateral	0.5–0.6	1.0–1.3
Single tip dorsal	0.4–0.8	1.3–1.7
Single lateral	0.6–0.8	
Double lateral	1.2	2.3
Double dorsal/ventral	0.7–1.3	2.2

Measurements of punctures (SEM)		
Average	0.98	1.9
Range	0.85–1.1	1.6–2.3
N	5	4

Stylet lengths (LM and SEM)		
(A) From pharynx to tip of infracapitulum (n = 16)		42.7 (39.3–46.8)
From tip of infracapitulum to stylet tip (n = 16)		86.7 (60–120)
(B) Entire stylet outside of infracapitulum (n = 8)		168.5 (135–193)
Working stylet (= B − A)		92–150
Stylet length (SEM) (n = 4)		76.1–110.7

**Fig. 2:**
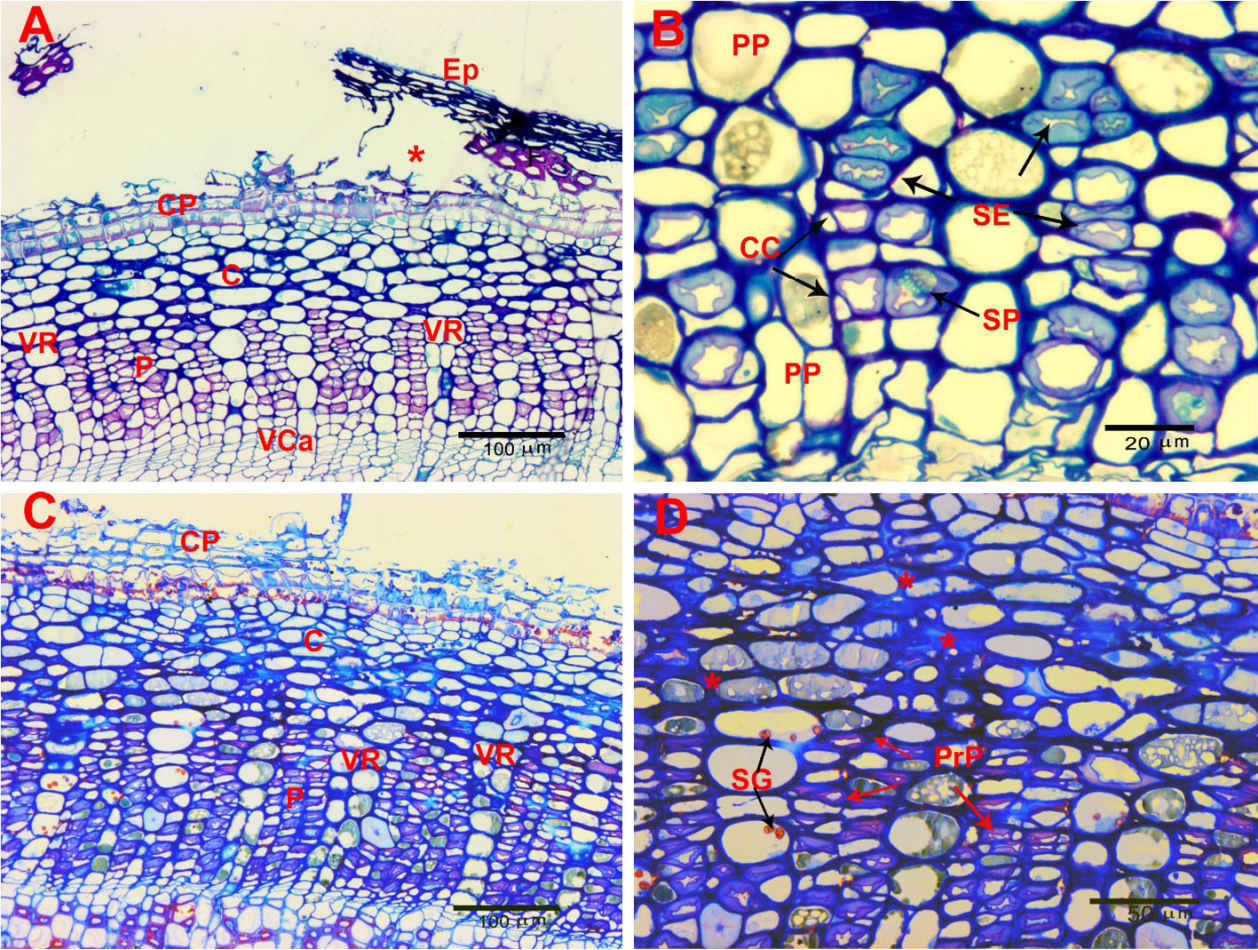
Light micrographs taken at 1 µm sections stained with methylene blue/azure A/basic fuchsin of control 1- and 2-year-old stems. **a** Cross section of part of 1-year-old stem under a fissure (asterisk). *Ep* epidermal layer, *CP* cork periderm, *C* cortex, *P* phloem, *VR* vascular ray, *VCa* vascular cambium. **b** Higher magnification of 1-year-old-stem showing. *SE* sieve elements, *PP* phloem parenchyma, *CC* companion cells. **c** Cross section of 2-year-old-stem in middle of fissure. Area labels as above. **d** Higher magnification of 2-year-old stem. *SG* starch grains, *PrP* collapsing protophloem sieve elements, red asterisks—areas of normal thickening of cortical primary cell walls

**Fig. 3:**
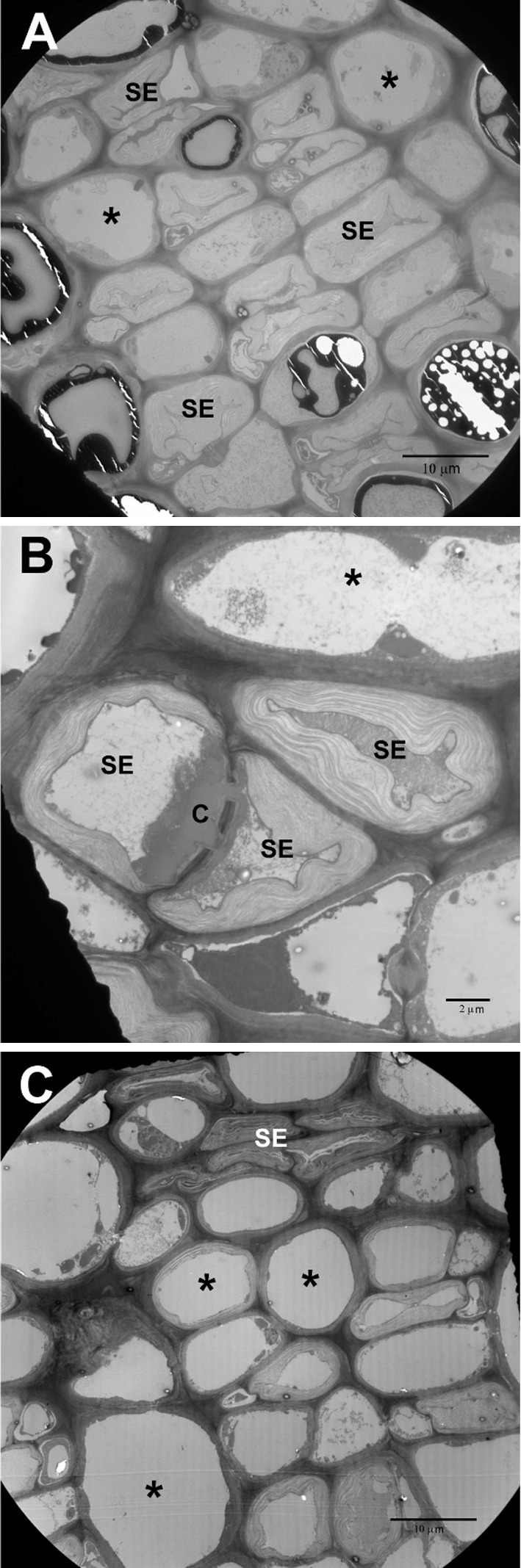
Transmission electron micrographs of control phloem in 1-year-old-stems. **a** *SE* sieve elements **b** Higher magnification of sieve elements showing thickened cell walls and lateral sieve plate filled with callose (C). **c** Lower magnification showing collapse of protophloem sieve elements. Asterisks in **a**, **b**, & **c** point to large central vacuoles of parenchyma cells

Figure 2 shows cross sections of healthy bark from young (1 year woody stems) and older (2–3 year woody stems) extending from the inner vascular cambium layer (VCa) to the outer developing cork periderm layer (CP). Figure 2a shows the edge of a crevasse with the outer cork layer and sloughed epidermal layer (Ep) creating a sheltered area in which *T. japonica* were frequently found (asterisks). In this young stem, the cork periderm is only 3 cell layers thick and overlays a cortical region (C) of 5–6 cell layers thick. Below the cortex is the area of active phloem (P) (Fig. 2b) consisting of thick walled sieve elements (SE), companion cells (CC) and phloem parenchyma (PP). Below the phloem layers is the vascular cambium (VCa) which, in this example, is 2–4 cells thick. Two points are to be noted: the orderliness of the arrangement of the cortical cells and the vascular rays (VR) and relative emptiness of the cortical cells and phloem parenchyma. The cells are not actually empty, the cytoplasm is simply pushed against the walls by a large central vacuole (asterisks) as shown by TEM in Fig. 3b, c. Because of this characteristic of the cortical cells and phloem parenchyma, finding evacuated cells caused by feeding injury was difficult with both LM and TEM. Figures 2b and 3a, b show the normal appearance of phloem sieve elements (SE) in these young stems.

Figure 2c, d show the normal arrangement of tissues in the bark of 2–3 year old stems. Note that the cork periderm and cortical layers look very much like the younger stems but with the exception of some thickening of the walls of the inner cortical cells (red asterisks) and the normal compression of the protophloem (PrP) sieve elements (the outer, original phloem elements laid down in the developing stem) caused by compression from the expansion of the active phloem layers (Fig. 3c). Note in Fig. 2c the persistent regularity of arrangement of the vascular rays (VR) and cortical cells.

**Fig. 4:**
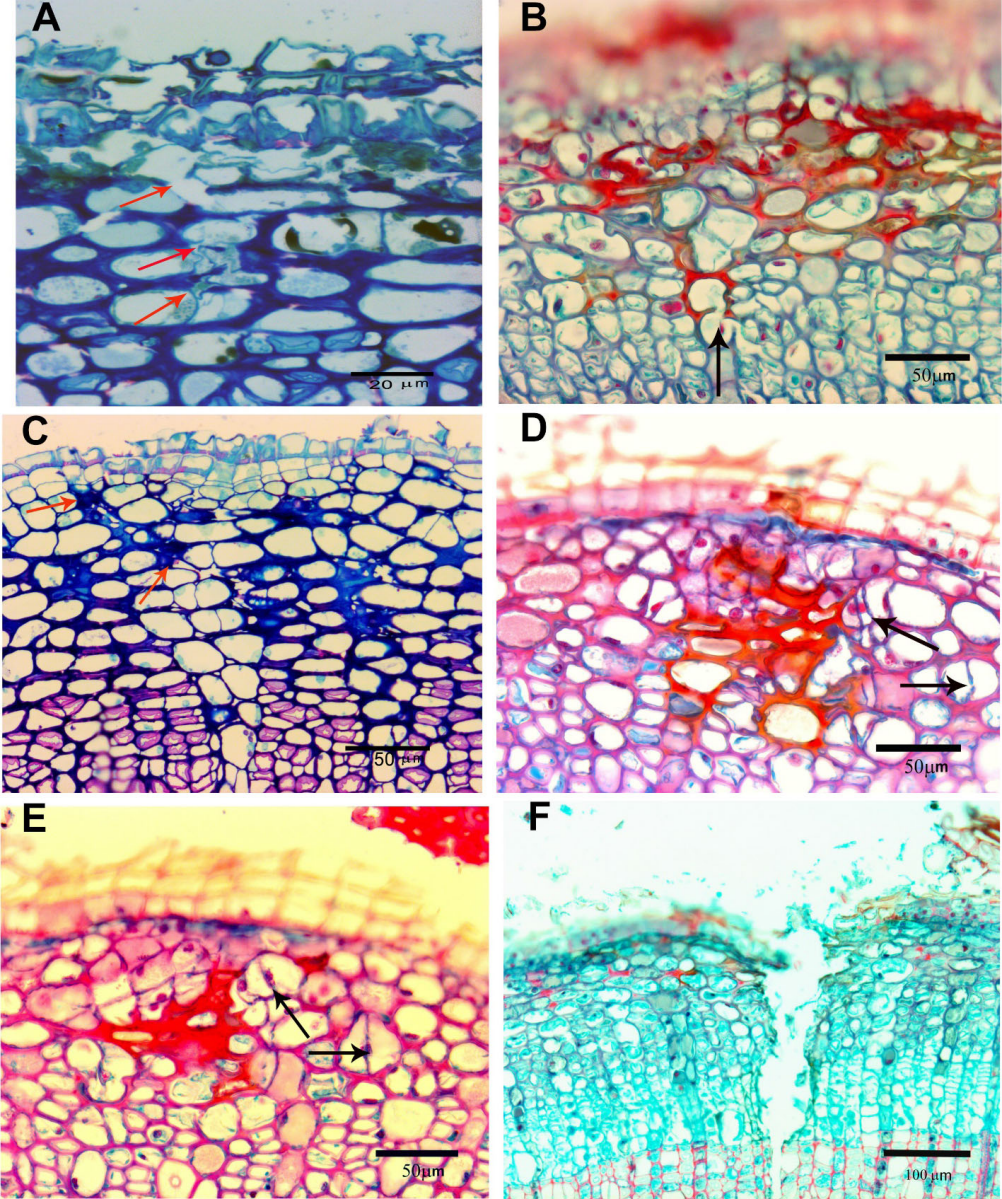
Light micrographs of stylet probing injury in *Camellia sinensis* cortical tissues. **a** 1-µm section with methylene blue/azure A/basic fuchsin. Arrow points to break in walls of cortical cells indicating passage of stylets. **b** 10-µm section stained with safranin/fast green. Dark red staining indicates lignification or presence of mite-injected saliva. Arrow points to break in wall from paired stylet passage. **c** 1-µm section stained as in **a**. Arrows point to possible path of stylets through cortical tissues. **d**, **e** 10-µm sections stained as in **b**. Red stain indicates presence of mite saliva. Arrows point to new cell walls, indication of cell division (hyperplasia) occurring with recovery from cell injury. **f** 10-µm section stained as in **b** of control puncture made with size 000 insect pin 3 days before sample was processed for microscopy injected along the stylet salivary tracks. Note lack of red staining in walls along the sides of the extensive puncture indicating lack of lignification from physical injury injected along the stylet salivary tracks

LM sections were examined for evidence of punctures or stylet tracks of collapsed cell walls in the tissue. Figure 4a, b (arrows) show some of the few areas of what appear to be disrupted cell walls caused by stylet punctures. In Fig. 4c, the arrows show what appears to be evidence of disruption of cells caused by a stylet-saliva path. The paraffin embedded tissue stained with Safranin and fast green (Fig. 4b, d, e) show conspicuously stained areas of what appear to be stylet-saliva tracks in the cortical layers. Safranin stains for the presence of lignin, suberin or saliva (Sass 1958). Specifically, safranin has been used to identify salivary tracks and sheaths produced by aphid and adelgid species (Pollard 1973; Young et al. 1995). In our case, we used it to distinguish between staining caused by salivary tracks and subsequent lignifications of affected tissues.

To determine whether the stylet tracks thus stained were from lignification or by-products of saliva injection, we punctured live stems with a 000-sized insect pin and looked for lignification. Figure 4f of a 3 day old puncture shows that our punctures did not result in the formation of lignin and suberin in the walls of the bordering disrupted cells. We concluded that the staining in the tissue was due to saliva proteins injected along the stylet feeding tracks.

**Fig. 5:**
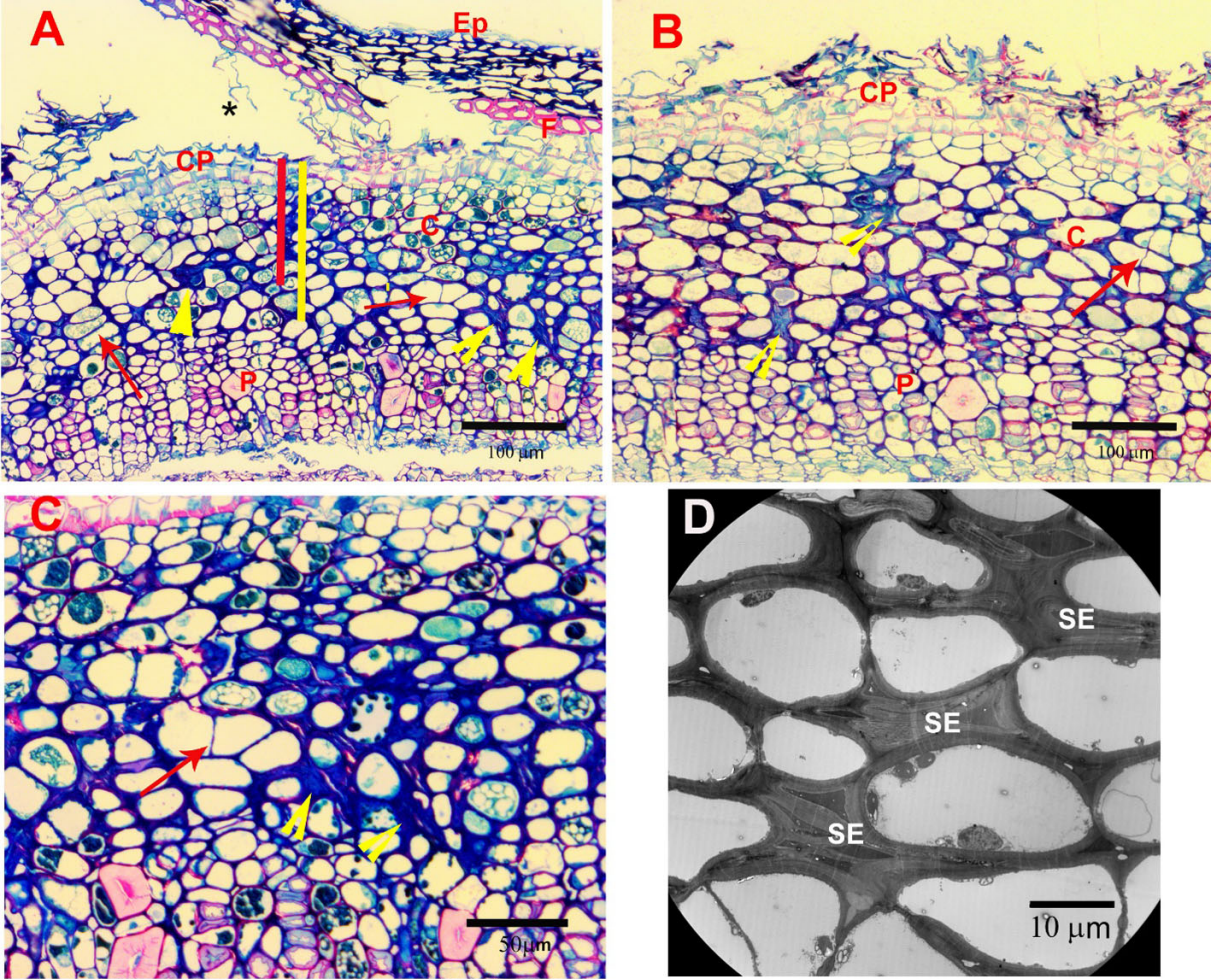
Light and TEM micrographs of advanced injury to stem. **a**, **b** Light micrographs of stem showing extensive disorganization of the cortical cells caused by mite saliva, resulting in collapsed cells (yellow arrowheads) and hyperplasia (red arrows). Red and yellow bars show the minimum (red) and maximum (yellow) range of stylet extension into the cortical cells (C). The maximum range extends into the outer layer of the phloem (P). *CP* cork periderm, *Ep* epidermal layer, *F* fibers, *crevasse. **c** Higher magnifications of **a**. **d** TEM micrograph showing extensive collapse of sieve elements (SE) in outer layers of phloem

Figure 4d, e (arrows) show evidence of newly divided cells (new cell walls) which is an indication of hyperplasia, an increase in the number of cells and a healing mechanism caused by wounded tissue. As previously mentioned, this is a common occurrence in spider mite damaged plant tissue. This type of recovery was also evident in the more severely affected tissue in Fig. 5a, b (red arrows).

**Fig. 6:**
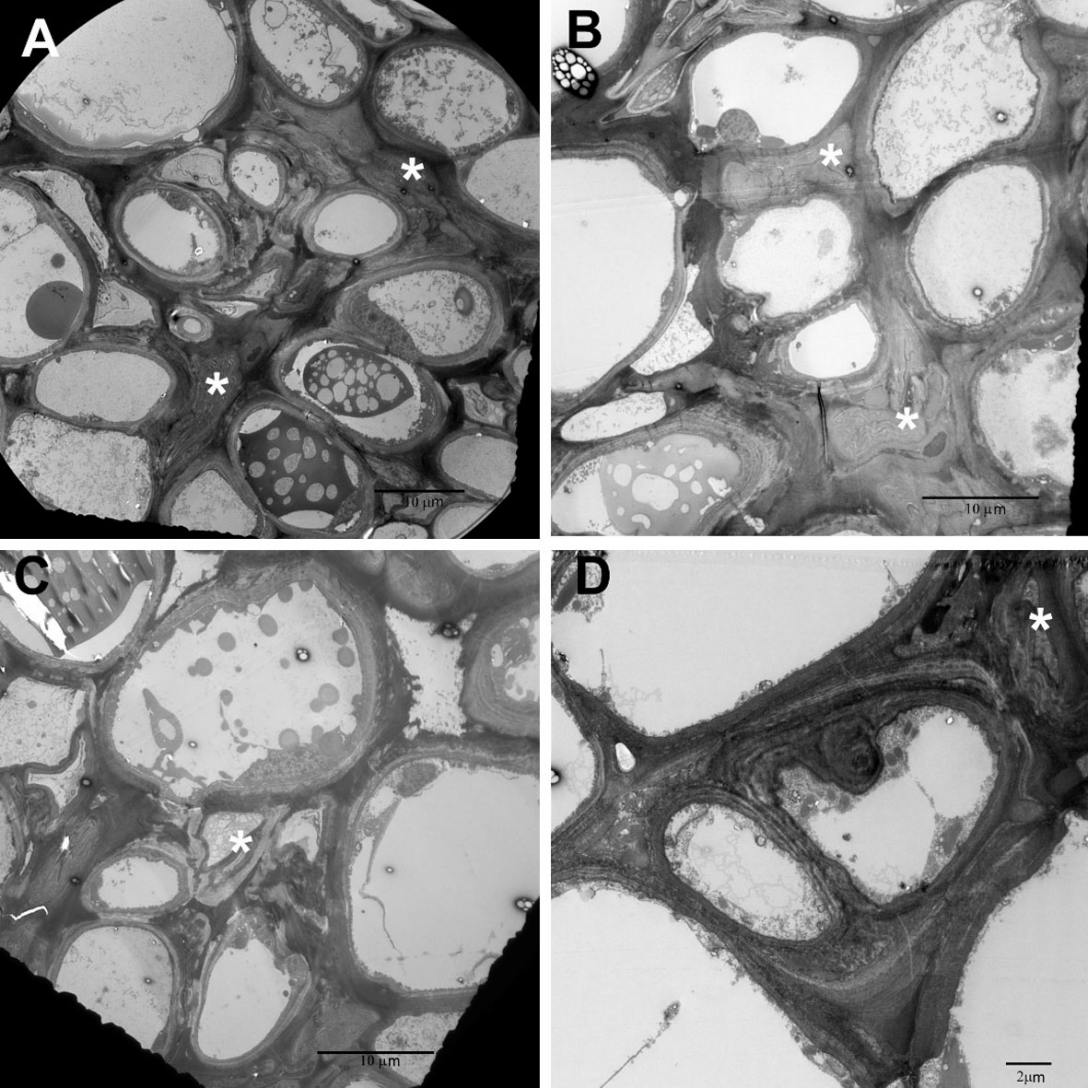
**a**–**c** TEM micrographs of high magnifications of Fig. 5 **a**–**c** showing the collapsed areas are actually collapsed cortical cells and collapsed sieve elements (white asterisks in **a**, **b**) rather than just thickening of cortical cell walls. **d** Two new sister cells (hyperplasia) occurring inside collapsed area

Figure 5a–c show the disorganization caused by more extensive injection of saliva in a 2-year old stem. Salivary stylet tracts of collapsed cells are evident (yellow arrowheads). Figure 6a–d are TEM images of those areas (asterisks) showing that they are collapsed cells rather than just thickened or lignified walls resulting from the aging of the tissue. Figure 6d shows two new sister cells from a cell division within a collapsed area of cells.

The question arose as to whether stylet penetration was confined to the cortical tissue or in the phloem or vascular cambium itself as suggested by Charles (2009). To help determine this, we measured the length of the stylets with both LM and SEM. We were unable to be certain that just measuring the stylet length that extended beyond the tip of the infracapitulum represented the full extent of its length. So we measured the entire stylet in those mites in which the stylets were not confined by the infracapitulum and subtracted from it the measurements that were made of the distance from the base of the infracapitulum to its tip. We felt that this gave us the full potential length available for feeding. From this we determined that the range of potential stylet penetration was from 92 to 150 µm. The vertical lines in Fig. 5a show that the outer layers of phloem tissue would be available to the mites stylets but not the vascular cambium. Figure 5d and 6a–c (asterisks) show evidence of collapsed sieve elements found in young tissue (Fig. 3c), older tissue (Fig. 5d), and in advanced injury (Fig. 6b) (asterisks). Whether the mites actually penetrated these upper sieve elements or the cells merely collapsed due to injection of saliva into nearby tissues is undetermined.

The stylets of *T. japonica* were measured with a coverslip pressing on the flattened mite. The flattened mite has a strong internal fluid pressure that pushes out the stylophore and the other appendages (de Lillo, pers. comm.). de Lillo suspects that the working range of stylets could be shorter for these reasons. Future studies should include using pieces of coverslip placed in the mounting medium to minimize risk of flattening the mite specimens as well as use of other methods to determine stylet lengths. Tuckerellid species need to be assessed for their potential to access phloem tissues in their host plants that are of economic importance.

Our conclusion is that *T. japonica* penetrates and injects saliva into the cortical tissue and possibly into the outer phloem tissue but not the vascular cambium in areas of bark exposed by splitting of the outer epidermis of 1- to 3+-year-old stems. Our evidence includes visible punctures, cellular collapse and cellular hyperplasia (wound healing) along obvious stylet-saliva tracks of the mites.
